# Beyond survival: psychoneuroimmunology-driven cancer psychological rehabilitation—a paradigm shift from symptom management to biobehavioral remodeling

**DOI:** 10.3389/fonc.2026.1942309

**Published:** 2026-09-16

**Authors:** Junjie Liu, Yinghao Shen, Jiangrui Liao, Xue Wang, Zhujun Mao, Yinlu Wang, Peng Zhang

**Affiliations:** 1 Wudang Mountain Branch, Taihe Hospital Affiliated to Hubei University of Medicine, Shiyan, China; 2 Graduate School, Harbin Sport University, Harbin, China; 3 School of Basic Medical Sciences, China Three Gorges University, Yichang, China; 4 College of Science and Technology, China Three Gorges University, Yichang, China; 5 School of Sports and Technology, Guangzhou College of Applied Science and Technology, Guangzhou, China; 6 Department of Rehabilitation Medicine, Longgang Central Hospital of Shenzhen, Wuhan, China

**Keywords:** biobehavioral remodeling, cancer psychological rehabilitation, immune reconstitution, precision intervention, psychoneuroimmunology

## Abstract

Cancer treatment paradigms have evolved from solely focusing on tumor eradication to optimizing survivorship trajectories. However, current psychological rehabilitation approaches predominantly target symptom relief, such as anxiety, depression, and fatigue, without direct evidence of influencing cancer’s biological processes like recurrence, metastasis, or immune evasion. This review, grounded in psychoneuroimmunology, advocates for elevating psychological rehabilitation from an auxiliary support role to a synergistic component of antitumor therapy. Central to this perspective is redefining rehabilitation endpoints to include immune reconstitution indices and tumor dormancy maintenance capacity. We propose a dynamic “stress-inflammation-immune escape” cycle model and emphasize the development of precision psychological interventions tailored to tumor genetic mutation profiles and associated psychological phenotypes. The article synthesizes three strands of recent evidence: First, clinical translational findings suggesting that psychological interventions may be accompanied by shifts in immunological indices such as the regulatory T cell (Treg)/T helper 17 (Th17) ratio and may correlate with the efficacy of PD-1 inhibitors, although the available evidence rests largely on small samples and surrogate endpoints and is insufficient to establish causality; Second, mechanistic findings, derived predominantly from preclinical models, indicating that the brain-bone marrow axis may mediate epigenetic reprogramming of hematopoietic stem cells, the human relevance of which remains to be confirmed; and finally, technological developments such as digital phenotyping that offer exploratory directions for monitoring relapse and psychological deterioration, whose diagnostic performance has not yet been established in prospective studies. By advancing the interdisciplinary field of precision psychoimmuno-oncology, this review aims to establish a low-cost, high-benefit “brain-body combined therapy” paradigm, offering cancer survivors innovative strategies for comprehensive recovery beyond mere symptom control. Importantly, the hypothesis framework proposed herein rests predominantly on surrogate endpoints and preclinical evidence. Until hard endpoints from prospective randomized controlled trials become available, any effect of psychological interventions on tumor biological behavior should be regarded as a scientific hypothesis awaiting verification rather than an established clinical conclusion.

## 1 Introduction

Cancer treatment is now an age where the aim of cancer treatment is not only survival, but to maximize every step of the cancer journey for the patient – including their health and quality of life (1). Conventional psychological rehabilitation plans for cancer patients have mostly been aimed at relieving patients’ symptoms including anxiety, depression, fatigue and so on (2, 3). While these interventions are critical to aid the patient, they do not take into account the intricate mechanisms by which psychological conditions, tumor biology, tumor microenvironment (TME) and immune evasion mechanisms interact (4). TME is a complex and dynamic environment that is composed of malignant cells, immune cells, stromal cells, a variety of extracellular matrices and soluble factors, and these components all play a crucial role in tumor progression, metastasis and resistance to therapy (5). The recent advances in the field of tumor biology have pointed out that psychological and social aspects are not only peripheral, but have an active role in the cancer process, through neuroendocrine and immune mechanisms (6). This recognition warrants a paradigm shift in cancer psychological rehabilitation from symptom management to a holistic approach to cancer that incorporates biobehavioral modulation strategies which attempt to alter the biology of a tumor (6, 7).

Psychoneuroimmunology (PNI) has become an important model that helps to explain the interactions between psychosocial stressors and the immune-endocrine-neural (IEN) system, particularly the hypothalamic-pituitary-adrenal (HPA) axis and the sympathetic nervous system (SNS) and their effects on tumor biology (8). Chronic stress, disruption of circadian rhythm, and social isolation (the so-called “psychological carcinogenic drivers”) have been reported to promote immunosuppressive effects and pro-inflammatory states leading to tumor immune escape, progression and metastasis (9, 10). For instance, activation of the HPA axis in response to stress increases the level of glucocorticoids that can inhibit the function of cytotoxic T cells and favor the expansion of regulatory T cells in the TME, thus reducing the anti-tumor immunity (5). Likewise, SNS activation results in the release of catecholamines which affect the trafficking of immune cells and the cytokine profiles, creating an immunosuppressive microenvironment that could drive tumor growth (11). These neuroimmune interactions highlight biological plausibility for the possibility that those psychological interventions that focus on stress reduction and circadian control may have real effects on tumor progression by influencing the immune environment (7, 8).

Importantly, however, there is a lack of transfer of these psychoneuroimmune findings into rehabilitation applications so far, which is a critical gap in the current treatment of cancer (12, 13). Conventional psychological support focuses mainly on emotional distress, and has no direct impact on the biological substrates of tumor progression (14, 15). A growing body of evidence, however, indicates that psychological interventions can serve as “epigenetic immunomodulators” that can “reprogram” immune responses and change patterns of gene expression of tumor and immune cells (16). Stress reduction, for example, social support improvement, and behavioral treatment can reestablish circadian rhythms and lessen chronic inflammation to break the vicious cycle of immune system suppression and tumor promotion. This biobehavioral reprogramming could be of benefit for synergistic combination with traditional anti-tumor treatments, thereby improving their effectiveness and decreasing the chances of cancers coming back (6, 8, 17, 18).

The tumor microenvironment is a hub where psychological, immune and metabolic factors all come together to affect the outcome of the cancer. Immune cells in the TME, including tumor-associated macrophages (TAMs) and myeloid-derived suppressor cells (MDSCs) and regulatory T cells are dynamically regulated by systemic neuroendocrine signals and local metabolic cues (19, 20). TME metabolic reprogramming also happens, such as lipid and amino acid metabolism, which further influences the functions of immune cells and tumor immune evasion (21, 22). These metabolic changes can be intensified by psychological stress and lead to an immunosuppressive environment. Therefore, psychological rehabilitation that incorporates metabolic and immune modulation represents a revolutionary approach to cancer care, transcending traditional symptom management to achieve biological remodeling of the tumor-host interface (6, 8).

To sum up, the future direction of cancer care will be toward facilitating the best possible survival curve using integrative psychological rehabilitation with the help of psychoneuroimmunology (23). In view of the broader concept of psychological support as “epigenetic immunomodulation,” there is a shift from symptom management to orchestration of a comprehensive biobehavioral reprogramming that targets the tumor microenvironment and immune escape mechanisms (6, 24). This paradigm revolution has the potential to synergize with other existing anti-tumor therapies, reduce recurrence and metastasis and ultimately improve survival and quality of life for cancer patients (25). The present review strives to communicate this novel paradigm by summarizing the existing literature regarding the complex interactions between psychological, neuroimmune and tumor biological aspects and by suggesting new approaches to a biologically informed psychological rehabilitation in oncology (7, 13). Importantly, oncology represents a profoundly heterogeneous field. As illuminated by recent advances in precision medicine, tumor entities differ not merely in their anatomical origins, but also exhibit fundamentally distinct biological behaviors driven by their underlying genomic and molecular characteristics (e. g., distinct molecular subtypes within the same organ). While the primary objective of this review is to establish an overarching conceptual framework for the integration of biomarkers and patient-reported outcome measures (PROMs) in general oncological rehabilitation, we will explicitly highlight and illustrate throughout the subsequent sections how these rehabilitation interventions and monitoring strategies must be further tailored and refined based on the specific genetic and molecular subtypes of the patients.

## 2 Methods: literature search strategy

### 2.1 Search strategy

To select eligible studies for this narrative review, an extensive literature search was performed in four electronic international databases: PubMed, Web of Science Core Collection, Scopus, and Embase. The primary search window covered publications issued from January 2020 to June 2026, with randomized controlled trials prioritized as the core evidence base given their superior methodological rigor. When mechanistic evidence or foundational theoretical support within this time frame was insufficient, high-impact classic studies and review articles published prior to 2020 were also considered for inclusion to preserve the integrity and theoretical depth of the overall argument. Search terms were organized into three key domains: cancer and tumor biology, psychoneuroimmunology and biobehavioral pathways, and psychological rehabilitation and interventions, and then combined using appropriate Boolean operators to search for literature that included both clinical translational and preclinical mechanistic studies. Reference lists of included articles and key review papers were also manually screened to complement the database search and minimize the risk of missing eligible studies.

### 2.2 Study selection

Two independent investigators, Junjie Liu and Xue Wang, completed the full literature screening process. Both investigators first screened titles and abstracts against predefined eligibility criteria to exclude clearly irrelevant records, followed by full-text assessment for potentially eligible studies. Studies were eligible for inclusion if they were peer-reviewed full-text articles published in English, focused on adult cancer populations, and explored either the association between psychosocial or behavioral factors and tumor immunity, tumor biology, or cancer prognosis, or the immunological and oncological effects of psychological interventions. Studies were excluded if they were case reports, conference abstracts, letters to the editor, study protocols without complete data, or studies that reported only quality of life, anxiety, or depression as sole endpoints without any immunological or tumor-related biomarkers. Disagreements between the two investigators arising during the screening process were resolved through in-depth discussion, with consultation from a third senior researcher when consensus could not be reached.

### 2.3 Limitations of this narrative review

As a narrative review rather than a pre-registered systematic review, this work carries inherent limitations related to evidence selection and interpretation (26, 27). Study inclusion was guided by the thematic framework of the review, and unavoidable subjective judgment was involved in evidence synthesis, which may introduce a tendency toward selective citation of positive findings; no quantitative meta-analysis was performed, so pooled estimates of intervention effect sizes cannot be provided (26). The heterogeneity of evidence in these included studies is high, as the cancer type, intervention modality, length of follow-up, and method of biomarker detection vary between studies, making it difficult to compare the results of studies directly or to draw conclusions that can be generalized to other tumor populations (14, 28). The majority of supporting evidence is based on surrogate immunological endpoints and a causal link between short-term immune modulation and long-term oncological outcomes still needs to be confirmed in large-scale prospective clinical trials (29). Furthermore, the fact that there is a bidirectional relationship between psychological status and tumor burden makes it challenging to exclude reverse causation from most of the existing trials, and most trials have been carried out in early breast and colorectal cancer populations in high-income areas; generalizability of results to advanced aggressive malignancies and low-resource settings is therefore limited (15, 30). Furthermore, most of the included studies employed observational or small pre-post designs and therefore cannot exclude reverse causation: tumor burden and treatment toxicity can themselves produce psychological distress and cognitive decline, generating non-causal covariation between psychological and immunological measures.

## 3 Redefining rehabilitation endpoints: from quality of life to immune reconstitution indices

### 3.1 Limitations of traditional endpoints

The traditional endpoints of rehabilitation in cancer care, such as QoL, depression score, and fatigue score, are mostly subjective and fall short of adequately reflecting the direct impact on the TME and immune surveillance functions (20, 31). Although important for understanding symptom burden and psychological well-being, these endpoints do not provide mechanistic insights into how psychological interventions might exert their effects on tumor biology or immune competence (6–8). Specifically, mindfulness-based stress reduction (MBSR) has been found to be effective in enhancing the emotional states and decreasing distress levels in cancer patients (including lung and breast cancer) in RCTs (32, 33). However, none of these changes in mood or QoL have been converted into statistically significant lower tumor recurrence rates, and there is a disconnect between subjective endpoints and objective oncological outcomes. This disconnect may be further compounded by molecular heterogeneity within the same tumor type. In breast cancer, for instance, hormone receptor-positive (Luminal) patients receiving endocrine therapy face distinct rehabilitation challenges, prominently including aromatase inhibitor-associated arthralgia and bone mineral density loss. Such side effects necessitate PROMs modules that specifically capture musculoskeletal symptoms and metabolic bone markers (34). By contrast, HER2-positive patients on anti-HER2 targeted therapy require systematic monitoring of cardiotoxicity, such as troponin surveillance, alongside regular fatigue assessment, as these dominate their treatment-related morbidity profile (35). Aggregating PROMs data across these biologically distinct subtypes without stratification risks obscuring subtype-specific signals and diluting the apparent QoL-survival association (36).

Similarly, in colorectal cancer, the distinction between microsatellite instability-high (MSI-H) or mismatch repair-deficient (dMMR) and microsatellite stable (MSS) tumors carries profound rehabilitation implications. MSI-H/dMMR patients, who derive substantial benefit from immune checkpoint inhibitors (ICIs) in metastatic settings, face a distinct toxicity profile dominated by immune-related adverse events (irAEs), including dermatitis, arthralgia, and endocrinopathies. By contrast, MSS patients treated with conventional chemotherapy contend primarily with neurotoxicity and myelosuppression. These divergent symptom trajectories demand subtype-specific PROMs instruments and rehabilitation protocols; pooling QoL data across MSI-H and MSS populations without stratification may obscure the true relationship between psychological well-being and oncological outcomes (37). In addition, a systematic review and meta-analysis further confirms that psychological interventions improve QoL domains (global, emotional, social and physical), but do not prolong overall or recurrence-free survival (29). This indicates that conventional endpoints might be insufficiently sensitive to reflect the complicated biological interplay between psychological status and tumor progression (38). Furthermore, patient-reported outcomes do not account for the complex and dynamic immunological and molecular changes that occur within the TME and are critical for tumor control (8, 20). Conventional QoL measures, for example, do not measure the modulation of neuroendocrine pathways that can result in immunosuppression and tumor-promoting inflammation as a consequence of chronic stress and depression (39, 40). A lack of biomarkers to assess immune function and/or the dormancy state of the tumor in rehabilitation endpoints leaves a mechanistic gap, limiting the ability to fully evaluate the effect of psychosocial interventions on cancer biology (7, 41). Furthermore, the diversity of psychological interventions and the different designs of the studies further complicate the interpretation of the effects of psychological interventions on survival outcomes (15, 29). Therefore, there is a pressing need to redefine rehabilitation endpoints beyond subjective symptom relief to include objective immunological and molecular indices that can elucidate the biological underpinnings of psychological interventions in cancer care (6, 41). This paradigm shift would help to more fully assess the effectiveness of therapeutic interventions, bridging the gap between symptom control and modification of biological behaviors in the TME (6, 23). These biomarkers could also be used on an individual basis to adjust psychosocial treatments that aim to regulate immune surveillance and tumor dormancy, with the aim of improving long-term oncological outcomes during clinical trials (42–44). In conclusion, traditional endpoints are important to evaluate patient well-being, but the limitations of these endpoints highlight the need for new biomarkers to evaluate the direct effects of psychological interventions on the dynamics of the tumor–immune system and drive progress in cancer rehabilitation from symptom management to mechanistic understanding and immune restoration (17).

### 3.2 Immune reconstitution indices as novel biomarkers

New research is beginning to provide evidence that immune reconstitution indices can be used as objective markers of the biological effects of psychological interventions in cancer rehabilitation, in addition to subjective symptom measures (17). The activity of natural killer (NK) cells is an important facet of innate immune surveillance against tumor cells. Psychological interventions can influence a number of neuroendocrine processes, including the quantity of cortisol, a glucocorticoid which has an inhibitory effect on NK cell function (45, 46). By reducing cortisol levels through stress management techniques, interventions enhance NK cell cytotoxicity, notably by increasing the expression of perforin on NK cell membranes, thereby improving tumor cell clearance efficiency (42, 47, 48). Wearable devices that are able to measure diurnal cortisol slopes provide a real-time, noninvasive surrogate measure of the efficacy of psychological interventions on hypothalamic-pituitary-adrenal (HPA) axis regulation and subsequent immune activation (49). A second key biomarker is the rate of T cell “exhaustion” reversal, especially for the immune checkpoint blockade drug PD-1 inhibitors (50, 51). The presence of high numbers of exhausted CD8+ T cells is associated with poor therapeutic response (52). Psychological interventions, including emotional expression writing, have been shown to inhibit regulatory T cell (Treg) differentiation, thereby alleviating immunosuppression and restoring CD8+ T cell functionality (53). The result of this immunomodulation is to increase the density of tumor-infiltrating lymphocytes (TILs) that have been shown to correlate with better control of tumors and better patient prognosis (42, 54, 55). Furthermore, the maintenance of tumor dormancy is a critical determinant of metastasis risk (56, 57). Psychological rehabilitation may also impact the epigenetic landscape of bone marrow hematopoietic stem cells (HSCs) to limit the formation of myeloid-derived suppressor cells (MDSCs) that have been shown to break tumor dormancy and activate metastases (58–60). Psychological interventions reduce the production of MDSCs, which help maintain the dormancy of these primary tumors and decrease the chance that micro-metastatic progression might happen (58, 61, 62). Collectively, immune reconstitution indices such as NK cell activity, reversal of T cell exhaustion, and maintenance of tumor dormancy represent candidate biomarkers that may reflect the biological effects of psychosocial interventions (43). It should be emphasized, however, that these indices currently serve as exploratory surrogate endpoints; their association with hard endpoints such as recurrence-free and overall survival has not been confirmed in prospective studies, and they should not be used in isolation as a basis for judging treatment efficacy (45). Their incorporation into the clinic would allow for dynamic monitoring of immune status and tumor biology, and thus provide a basis for personalized rehabilitation approaches to restore the immune system (8, 41, 45). This is consistent with the shift in paradigm from symptom-based endpoints to mechanistic biomarkers for a more holistic framework to assess and optimize psychological rehabilitation in cancer (17, 23, 41). Future work should be directed toward validation of these biomarkers in other cancer cohorts and toward standardization of measuring these markers – such as wearable cortisol monitors and immunophenotyping assays (10, 49). Finally, immune reconstitution indices show promise as objective, biologically relevant endpoints which could link psychological well-being and tumor immunology and lead to the ultimate goal of a precision medicine approach to cancer rehabilitation (**Table 1**) (8).

**Table 1:** Core biomarkers of immune reconstitution for psychoneuroimmunology-driven cancer psychological rehabilitation.

Biomarker	Biological function	Regulatory pathway of psychological intervention	Detection assay	Clinical application value	Reference
NK cell cytotoxic activity	Core effector of innate immune surveillance, directly eliminates circulating tumor cells and micrometastatic foci	Reduces cortisol level and upregulates perforin expression on NK cell membranes	Peripheral blood flow cytometry/lactate dehydrogenase release assay	Evaluates innate immune reconstitution and predicts tumor dormancy status	(45, 63, 64)
CD8^+^ T cell exhaustion reversal rate	Reflects the recovery degree of adaptive anti-tumor immune function	Inhibits Treg differentiation and increases tumor-infiltrating lymphocyte (TIL) density	Immunohistochemistry/flow cytometry for PD-1^+^Tim-3^+^ CD8^+^ proportion	Predicts therapeutic response to immune checkpoint inhibitors	(65–67)
Treg/Th17 cell ratio	Core homeostatic indicator balancing immunosuppressive and pro-inflammatory immunity	Mindfulness-based interventions downregulate Treg proportion and correct immune deviation	Peripheral blood lymphocyte subset immunophenotyping	Assesses immunosuppressive severity of the tumor microenvironment	(68–70)
Myeloid-derived suppressor cell (MDSC) proportion	Key myeloid subset mediating immune escape and disrupting tumor dormancy	Reverses epigenetic reprogramming of hematopoietic stem cells and reduces myeloid differentiation bias	Flow cytometry for CD11b^+^CD33^+^HLA-DR^-^ cells in peripheral blood	Predicts distant metastasis risk and long-term recurrence probability	(61)
Tumor dormancy maintenance capacity	Reflects the stability of micrometastatic foci in a quiescent state	Reduces pro-inflammatory cytokine levels and inhibits angiogenic switch activation	Dynamic ctDNA monitoring + circulating tumor cell viability assay	Core biological surrogate endpoint for recurrence-free survival	(56, 57, 71)
Diurnal cortisol slope	Core biomarker of HPA axis homeostasis, indirectly reflects immune regulatory status	Stress management and sleep interventions restore physiological cortisol rhythm	Continuous interstitial fluid/sweat cortisol monitoring via wearable devices	Real-time evaluation of neuroendocrine efficacy of psychological interventions	(8, 9, 72, 73)

Within the regulatory cascade depicted here, the H3K4me3 modification changes and the myeloid bias are supported principally by mouse models, whereas the remaining steps derive from human observational studies; the entire cascade represents a mechanistic hypothesis rather than a confirmed causal pathway. This table compiles objective immunological biomarkers that supplement traditional subjective quality-of-life endpoints and allow quantitative characterization of the tumor-immune dynamics accompanying psychological interventions. It should be noted that evidence for most indices listed here derives from small pre-post studies or preclinical models; they should therefore be regarded as exploratory surrogate endpoints rather than validated predictive biomarkers, and their cutoff values and assay methods have not yet been standardized.

### 3.3 Epigenetic immune regulation as the mechanistic foundation

Epigenetic immune regulation represents the mechanistic basis for understanding the interaction between psychological state and immune function in cancer rehabilitation and is a critical “epigenetic switch” that regulates the switch from immune exhaustion to immune reconstitution (74–76). Chronic psychological stress induces activation of β-adrenergic signaling pathways that activate the nuclear factor kappa-light-chain-enhancer of activated B cells (NF-κB) pathway in hematopoietic stem cells (HSCs) (58). This activation biases HSC differentiation toward myeloid lineages, resulting in increased production of immunosuppressive cells such as myeloid-derived suppressor cells (MDSCs) and regulatory T cells (Tregs), thereby fostering an immunosuppressive tumor microenvironment that facilitates tumor progression (42, 77, 78). At the chromatin level, stress-induced glucocorticoids engage the glucocorticoid receptor (GR), which binds to specific chromatin loci to enhance transcription of pro-inflammatory genes, including interleukin-6 (IL-6) and tumor necrosis factor-alpha (TNF-α) (79, 80). This epigenetic modulation sustains a chronic inflammatory environment which negatively affects effective anti-tumor immunity (8, 81). However, hyperactivation of the HPA axis can be reversed with short-term psychological interventions, leading to a reduction in GR binding to chromatin and a reduction in pro-inflammatory gene expression, thus restoring immune homeostasis (8, 9, 12). This bidirectional epigenetic regulation highlights the ability of psychological rehabilitation to act as a molecular “switch” from a state of exhaustion and suppression to reactivation and surveillance of the immune system (41, 74). As seen in new models, novel conceptual frameworks envision psychological interventions as epigenetic modulators that alter immune phenotypes at the molecular level to change clinical endpoints from subjective symptom scores to measurable molecular immune signatures (42, 82). This paradigm changes the concept of cancer rehabilitation because it is based on the interaction of psychosocial factors with molecular immunology and focuses on the importance of epigenetic phenomena as the mediators of the biological effects of psychological health on tumor immunity (44). Future studies should involve strategies to define unique epigenetic modifications and/or chromatin remodeling events that can be modified through psychological interventions, including chromatin immunoprecipitation sequencing (ChIP-seq) and single-cell epigenomics. Moreover, the combination of such epigenetic marks and immune phenotyping as well as clinical outcomes will help design targeted psychosocial treatments to optimize immune reconstitution. In summary, epigenetic immune regulation provides the mechanistic cornerstone for a transformative approach to cancer psychological rehabilitation, enabling a shift from symptom management to biological behavior remodeling through modulation of immune cell differentiation and gene expression patterns (**Figure 1**) (76, 83). It is important to state explicitly that the evidence underpinning this epigenetic cascade is not uniform. That chronic stress activates NF-κB via β-adrenergic signaling and thereby skews hematopoiesis toward the myeloid lineage derives principally from mouse models and *in vitro* experiments, and therefore constitutes preclinical evidence. That glucocorticoid receptor binding to chromatin alters pro-inflammatory gene transcription has been partially verified in human peripheral blood cells, and therefore constitutes human observational evidence. By contrast, the proposition that short-term psychological interventions can reverse HPA axis hyperactivation and restore immune homeostasis is currently supported only by small-scale pre-post studies, whose effect sizes and reproducibility remain to be confirmed, and thus represents preliminary clinical evidence. Accordingly, we position epigenetic immune regulation as a candidate mechanistic hypothesis explaining the psyche-immune association rather than as a demonstrated causal pathway; temporal and dose-response relationships obtained in animal models should not be extrapolated directly to clinical practice.

**Figure 1:**
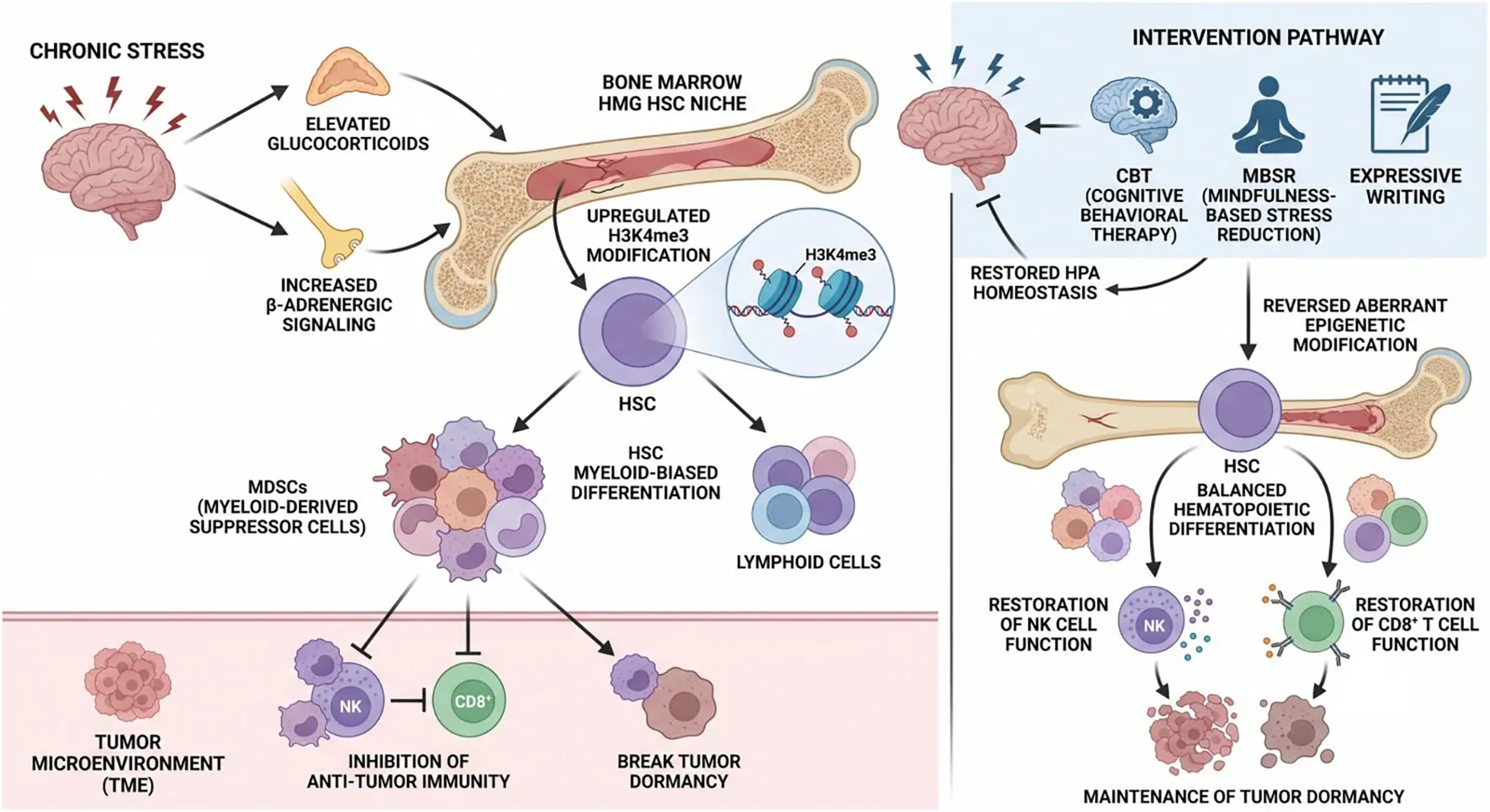
Epigenetic reprogramming of the brain–bone marrow axis mediates psychological modulation of anti-tumor immunity.

**Figure 1** depicts bidirectional epigenetic regulation of anti-tumor immunity mediated by the brain–bone marrow axis. Chronic stress elevates stress hormones and induces abnormal H3K4me3 modification in bone marrow HSCs, skewing hematopoiesis toward myeloid lineages and generating abundant MDSCs to disrupt tumor immune surveillance. Psychological interventions can restore the balance in immune cell differentiation, reverse HSC epigenetic reprogramming, restore the function of cytotoxic lymphocytes and preserve tumor dormancy. Abbreviations: HSCs, hematopoietic stem cells; H3K4me3, histone H3 lysine 4 trimethylation; MDSCs, myeloid-derived suppressor cells; CBT, cognitive behavioral therapy; MBSR, mindfulness-based stress reduction; HPA, hypothalamic-pituitary-adrenal.

## 4 Dynamic biobehavioral model: “stress-inflammation-immune escape” cycle and real-time interruption

### 4.1 Components and driving factors of the cycle model

Chronic psychological stress is a key initiating factor in the dynamic biological behavior model linking stress, inflammation, and immune escape in the progression of cancer. HPA axis activation in response to chronic stress results in the production and release of glucocorticoids like cortisol, and catecholamines like norepinephrine, which have direct effects on the biology of the tumor (9, 84). For example, in human oral squamous cell carcinoma (OSCC) cells, stress hormones such as norepinephrine and cortisol have been proven to significantly increase the secretion of interleukin-6 (IL-6) and promote tumor cell proliferation through the β-adrenergic receptor signaling pathway (8, 63, 80, 85). Thus, studies have demonstrated that exposure to norepinephrine at physiological levels of stress results in a large increase in the expression of IL-6 mRNA and the production of IL-6 protein, which can be inhibited by the use of β-adrenergic antagonists confirming the receptor-mediated mechanism (86, 87). Stress hormones activate NF-κB and STAT3 pathways, fostering a pro-tumorigenic microenvironment through the secretion of IL-6 and vascular endothelial growth factor (VEGF), both of which are critical promoters of tumor growth and angiogenesis (85, 88). The inflammatory cascade is further amplified because IL-6 can stimulate the liver to produce acute-phase proteins such as C-reactive protein (CRP), thereby increasing systemic inflammatory markers and exacerbating immune dysfunction (88). Elevated CRP levels are associated with decreased T cell function, such as T cell exhaustion and reduced T cell cytotoxic activity, which in turn leads to weakened host anti-tumor immunity (89–91). The prognostic weight of such inflammatory markers, however, is not uniform across breast cancer subtypes. In triple-negative breast cancer (TNBC), a subtype lacking targetable driver mutations, elevated preoperative interleukin-6 (IL-6) and C-reactive protein (CRP) levels have been identified as independent prognostic factors for overall survival, with one study reporting a hazard ratio of 11. 739 for the combination of elevated IL-6 and TNBC subtype. By contrast, the predictive value of CRP appears more modest in hormone receptor-positive tumors. This subtype-dependent prognostic significance suggests that inflammatory biomarkers may be particularly informative for rehabilitation monitoring in TNBC patients, where conventional targeted therapies are limited and immunologic resilience becomes a critical determinant of outcome (92, 93). This systemic inflammation promotes the recruitment and expansion of immunosuppressive cell populations in the tumor microenvironment, such as myeloid-derived suppressor cells (MDSCs) and regulatory T cells (Tregs) (94, 95). These cells are able to inhibit the activity of CD8+ cytotoxic T lymphocytes and natural killer (NK) cells, thereby providing an immune escape loop that enables tumor cells to evade immune surveillance and promotes metastasis (5, 96). Chronic stress-induced neuroendocrine signaling, inflammatory cytokine production, and modulation of immune cells are present in a self-sustaining cycle that promotes tumor progression. This model highlights the need to address the stress-inflammation-immune axis to break the vicious cycle of tumor-promoting inflammation and immune evasion (**Table 2**).

**Table 2:** Psychogenic carcinogenic drivers and targeted intervention nodes in the “stress-inflammation-immune escape” cycle.

Psychogenic carcinogenic driver	Core neuroendocrine signaling pathway	Downstream tumor biological effects	Targeted intervention strategy	Level of evidence	Reference
Chronic psychological stress	Sustained HPA axis activation + sympathetic nervous system β-adrenergic signaling	Activates NF-κB/STAT3 pathways, upregulates IL-6 and VEGF, promotes tumor proliferation and angiogenesis	Mindfulness breathing training + real-time biofeedback-assisted stress reduction	Grade A (supported by randomized controlled trials)	(6, 8, 80)
Circadian rhythm disruption	Aberrant melatonin secretion and dysregulated clock gene expression	Decreased NK cell cytotoxicity, chronic low-grade inflammation, impaired hematopoietic homeostasis	Chronobiological light exposure intervention + exogenous melatonin supplementation	Grade B (supported by cohort studies and mechanistic validation)	(97–99)
Social isolation and loneliness	Anterior cingulate cortex activation and upregulated pro-inflammatory cytokine transcription	Reduced CD8^+^ T cell tumor infiltration and impaired immune surveillance	Online peer support groups + VR-based interactive social intervention	Grade B (supported by clinical observations and mechanistic studies)	(100–102)
Persistent depressive state	Blunted HPA axis negative feedback and sustained glucocorticoid elevation	Impaired cytotoxic T cell function, expanded Treg population, enhanced immune evasion	Cognitive behavioral therapy (CBT) + emotional expressive writing	Grade A (supported by systematic review evidence)	(10, 23, 41, 81)
Trait anxiety disorder	Elevated sympathetic tone and sustained catecholamine release	Promotes tumor cell migration and invasion, increased MDSC recruitment	Mindfulness-based stress reduction (MBSR) + progressive muscle relaxation	Grade B (supported by small-sample RCTs and mechanistic studies)	(13, 103, 104)
Post-traumatic stress symptoms	Hyperactive locus coeruleus-norepinephrine system and amplified inflammatory response	Elevated systemic inflammatory cytokine profile and epigenetic reprogramming of hematopoietic stem cells	Trauma-focused cognitive processing therapy + expressive writing	Grade C (supported by clinical observations and preliminary experimental data)	(105–107)

This table delineates the complete causal cascade from psychosocial risk factors to tumor immune evasion, and matches each driver with evidence-based intervention strategies to interrupt the pathological cycle.

### 4.2 Mechanisms targeting “psychogenic carcinogenic drivers”

Interventions to disrupt the psychogenic contribution to cancer progression are based on real-time monitoring and modulation of physiological parameters related to stress (49, 108, 109). It is possible to monitor chronic stress objectively by wearing cortisol monitoring devices, which include sweat or interstitial cortisol monitoring smart wristbands that can detect cortisol changes (110). Such devices that are combined with algorithms in affective computing can be used to identify periods of cortisol peaks that signal increased activity of the HPA axis. Upon detection of such peaks, personalized mindfulness-based breathing exercises can be triggered to attenuate stress responses and prevent excessive neuroendocrine activation, thereby blocking the downstream pro-tumorigenic signaling cascade (111, 112). Social isolation, a very strong psychosocial stressor, stimulates the anterior cingulate cortex (ACC), which further stimulates pro-inflammatory cytokines leading to systemic inflammation, and immune system suppression (97). A sleep intervention using chronobiology (i. e., timed light exposure and melatonin supplementation) can restore natural circadian rhythm, increase innate immune surveillance and decrease tumor-promoting inflammation (113). Social isolation, a potent psychosocial stressor, activates the anterior cingulate cortex (ACC), which in turn promotes the release of pro-inflammatory cytokines, exacerbating systemic inflammation and immune suppression (16). Digital social network analysis can be used to identify patients at high risk of isolation, by measuring social connectivity metrics. These patients can be matched to online peer support groups, including virtual reality (VR)-based interactive platforms, which have demonstrated efficacy in reducing loneliness and inflammatory markers by fostering social engagement and emotional support (102, 114, 115). Collectively, these targeted strategies address the psychogenic drivers of cancer progression by modulating neuroendocrine stress responses, restoring circadian homeostasis, and enhancing social support, thereby interrupting the stress-inflammation-immune escape cycle (23).

### 4.3 Real-time biobehavioral feedback systems to interrupt the cycle

The combination of multimodal physiological monitoring technologies provides the foundation for the development of real-time biobehavioral feedback systems to break the cycle of stress-inflammation-immune escape in cancer patients (116, 117). By combining continuous wearable sensors that measure cortisol levels, electrodermal activity (EDA), and heart rate variability (HRV), individualized psychophysiological baseline models can be constructed (49, 99, 118). These models incorporate the dynamic interaction between neuroendocrine and autonomic nervous system activity, and offer sensitive markers for imminent stress responses (111). Advanced machine learning algorithms analyze these multimodal data streams to detect early warning signs of stress-induced immune dysregulation, such as a decline in HRV coupled with rising cortisol levels minutes before a stress episode (119–121). This predictive ability allows interventions (such as guided relaxation of the autonomic nervous system through techniques such as paced breathing or biofeedback) to be carried out in time to prevent immune suppression from occurring as a result of stress (23, 111, 122). Randomized controlled trials have been used to show clinical translation of this approach, with a randomized controlled trial showing increased NK cell activity in cancer patients after eight weeks of using a real-time feedback system and a significant reduction in the level of cortisol during the night (p=0. 03) (116), a marker of immune and stress regulation. These are examples of a paradigm shift from a reactive symptom management approach to an active biobehavioral modulation approach that uses technology to personalize mental and physical interventions (123, 124). This strategy has the potential to increase cancer patients’ resilience by disrupting the harmful cycle of chronic stress, inflammation, and immune evasion, thereby boosting their clinical outcomes and quality of life (**Figure 2**).

**Figure 2:**
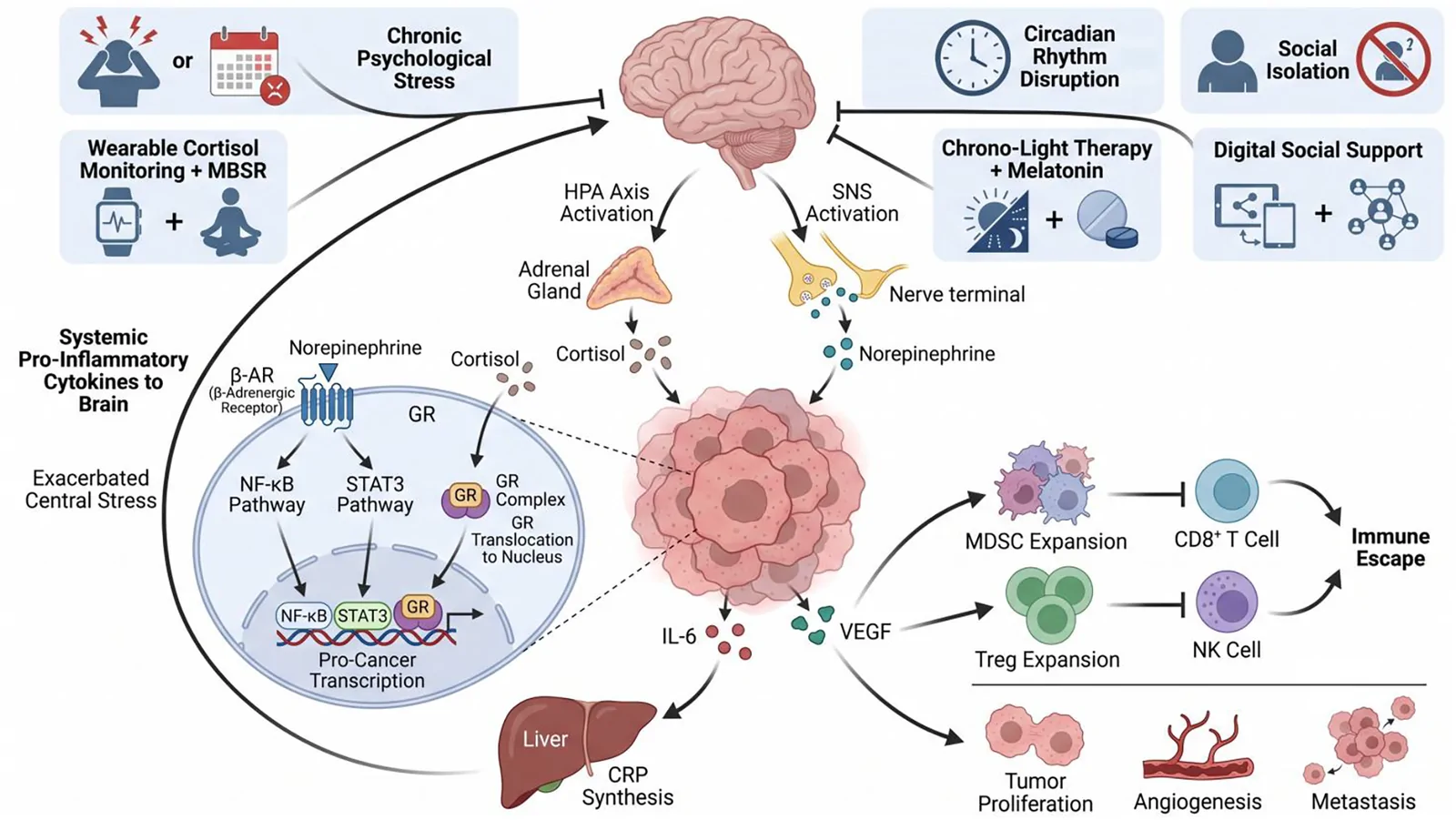
Molecular mechanism of the stress–inflammation–immune escape cycle and real-time biobehavioral intervention nodes.

**Figure 2** illustrates the vicious stress–inflammation–immune escape cycle and corresponding real-time biobehavioral intervention nodes. Chronic psychological stress, circadian disruption, and social isolation activate HPA and SNS signaling to release cortisol and norepinephrine. The two hormones separately act on cytoplasmic GR and membrane β-AR to trigger NF-κB and STAT3 transcriptional cascades. Tumor cells secrete IL-6 and VEGF to raise systemic CRP, expand MDSCs and Tregs, and suppress CD8^+^ T and NK cell function, driving tumor progression and distant metastasis. Circulating pro-inflammatory cytokines feed back to the central nervous system to amplify stress signals. Three behavioral interventions block upstream pathological drivers at distinct entry points. Abbreviations: HPA, hypothalamic-pituitary-adrenal; SNS, sympathetic nervous system; GR, glucocorticoid receptor; β-AR, β-adrenergic receptor; NF-κB, nuclear factor κB; STAT3, signal transducer and activator of transcription 3; IL-6, interleukin-6; VEGF, vascular endothelial growth factor; CRP, C-reactive protein; MDSCs, myeloid-derived suppressor cells; Tregs, regulatory T cells; MBSR, mindfulness-based stress reduction.

### 4.4 Real-time biobehavioral feedback systems to interrupt the cycle

It must be acknowledged that the association between psychological state and cancer is not unidirectional. On the one hand, chronic psychological stress can promote tumor progression via the HPA axis and the sympathetic nervous system: preclinical work has shown that, in a chronic unpredictable mild stress (CUMS) model, depressive-like behavior is accompanied by accelerated breast cancer growth, and that this pro-tumor effect can be reversed by modulating glutamate metabolism in the gut microbiota (125). On the other hand, the tumor itself and its treatment can impair psychological and neurological function in the reverse direction: systemic inflammation associated with the tumor-bearing state can compromise blood-brain barrier integrity, allowing chemotherapeutic agents to enter the brain more readily and inducing pyroptosis and ferroptosis of hippocampal neurons, which manifests clinically as ‘chemobrain’ and related cognitive and affective disturbances (126).

This bidirectionality has two methodological consequences. First, the direction of causality cannot be determined from psychological-immune associations observed in cross-sectional studies: a psychological measure may be either a cause or a consequence of immunological change. Second, anti-neoplastic treatment itself alters both psychological state and immunological indices, thereby acting as a confounder; estimates of the psychological-immune association will be biased unless tumor burden and treatment intensity are measured and adjusted for simultaneously.

Accordingly, the ‘stress-inflammation-immune escape’ cycle proposed here should be understood as a dynamic system containing feedback loops rather than a unidirectional linear pathway. Future studies should adopt prospective designs with cross-lagged analysis and should prespecify tumor burden and treatment exposure as covariates, in order to separate the main effect of psychological factors on tumor biology from the reverse effect of tumor burden on psychological state.

## 5 Precision psychological intervention: individualized prescription based on genes and psychological phenotypes

### 5.1 Association between tumor gene mutation characteristics and psychological phenotypes

The complex relationship between molecular tumor characteristics and psychological traits opens a new field of precision psycho-oncology, allowing for the development of individual psychological treatments, tailored to molecular tumor characteristics (127, 128). TP53 mutations are among the most studied mutations and are often correlated with a high inflammatory phenotype with high levels of interleukin-6 (IL-6) (68, 129, 130). This pro-inflammatory milieu predisposes patients to chronic stress-induced activation of the nuclear factor kappa-light-chain-enhancer of activated B cells (NF-κB) pathway, a key regulator of immune and inflammatory responses (6, 131). Chronic NF-κB activation not only fosters the progression of tumors, but also leads to increased psychological stress, with increased anxiety and depressive symptoms. Hence, mindfulness-based interventions like Mindfulness-Based Stress Reduction (MBSR), which have been shown to be able to decrease NF-κB signaling and reduce systemic inflammation (6, 82), may be advantageous to patients with TP53-mutated tumors. This targeted approach is consistent with new research showing how mindfulness interventions can alter neuroimmune pathways to reduce negative impacts of chronic stress on tumor biology and psychological health (132).

In contrast, KRAS mutations have been associated with elevated oxidative stress and DNA damage within tumor cells that ultimately result in a unique psychological profile that includes pathological worry and cognitive inflexibility (11, 133). The resulting increase in cortisol levels, due to persistent worry, adds to the oxidative damage and hampers immune surveillance. Cognitive flexibility enhancement, such as cognitive-behavioral therapy (CBT) (134), should thus be a focus of psychological rehabilitation for patients with KRAS-mutated tumors. CBT’s structured approach allows for cognitive restructuring, which helps patients to handle the maladaptive worry and decrease hyperactivity of the hypothalamic-pituitary-adrenal (HPA) axis, which in turn decreases cortisol levels. Such neuroendocrine modulation could result in changes in the tumor microenvironment and outcomes (9, 135). Notably, the psychosocial vulnerability of KRAS-mutant patients may extend beyond cognitive inflexibility to encompass heightened susceptibility to stress-induced immune dysregulation. KRAS mutations drive immune suppression in colorectal cancer through multiple mechanisms, including the recruitment of myeloid-derived suppressor cells (MDSCs) via the KRAS-IRF2-CXCL3-CXCR2 axis. Given that chronic psychological stress itself promotes MDSC mobilization and systemic inflammation, KRAS-mutant patients may face a synergistic immunosuppressive burden when exposed to psychosocial stressors. This hypothesis warrants prospective investigation and, if confirmed, would support genotype-informed psychosocial support strategies. Additionally, KRAS mutations have been associated with higher depression severity in older metastatic colorectal cancer patients, independent of gender and performance status signaling (136).

In addition, a positive psychological phenotype, called post-traumatic growth (PTG), is represented by an enhancement of the capacity of cognitive restructuring and an enhancement of emotional processing capacities (137, 138). Patients with high PTG tendency are resilient and have adaptive coping strategies, which could be considered therapeutic targets in expressive writing interventions (138, 139). Expressive writing facilitates emotional disclosure and cognitive processing of cancer-related trauma, promoting psychological adjustment and potentially influencing immune function. Because of the important role of natural killer (NK) cells in the immunosurveillance of tumors, monitoring their activity could be useful as a biomarker of response to treatment in these patients. Enhanced NK cell cytotoxicity correlates with improved psychological states and may reflect the biological impact of psychological interventions (140). All these associations highlight the importance of combining tumor genetic profiles with psychological phenotyping to tailor psycho-oncological care to the individual, thus connecting molecular oncology and behavioral medicine in a precision medicine approach (139, 141, 142).

### 5.2 Framework for designing targeted psychological prescriptions

Developing a robust framework for targeted psychological prescriptions necessitates a multidimensional stratification model that integrates tumor genetic mutations, psychological phenotypes, and immune status to guide intervention selection (42, 143). A conceptual decision tree may be used to operationalize this approach and classify patients according to the presence of major genetic changes (TP53 and KRAS mutations), psychological characteristics (anxiety sensitivity and cognitive flexibility), and immunological markers (regulatory T cell (Treg) percentage and T cell exhaustion score). This stratification allows clinicians to match patients to the best psychological and pharmacological treatments, increasing the efficacy of the treatments and personalization (144).

A combined MBSR/β-adrenergic blockade (propranolol, etc.) intervention is recommended for patients with a high-inflammatory and high-anxiety profile, who have high levels of IL-6 and psychological distress. This dual action is directed toward both inflammatory and stress signaling pathways, which involve NF-κB and catecholamines, respectively, leading to both reduced pro-tumorigenic signaling cascades and alleviation of psychological symptomatology (63, 145). The interaction between MBSR and β-blockers is a good example of a biobehavioral intervention that can affect the interactions between neuroimmune systems and can therefore enhance the control of tumors and patients’ quality of life (132).

On the other hand, patients with low inflammatory markers who have low social support would benefit from interventions that increase social support, including online social support groups. Enhancement of the social support network has been demonstrated to enhance CD8+ T cell infiltration into the tumors through intercellular adhesion molecule-1 (ICAM-1)-mediated extravasation, increasing the density of tumor-infiltrating lymphocytes (TILs) and anti-tumor immunity (54). This immunomodulatory effect is an important finding because it shows how important psychosocial factors are to the tumor microenvironment and lends further support to the inclusion of social support enhancement in individualized psychological prescriptions (146).

Another key aspect of this framework is temporal optimization of psychological interventions. Targeted psychological support within specific timeframes – within 48 hours before and 72 hours after immune checkpoint inhibitor (ICI) treatment – capitalizes on this ‘immune cycle amplification effect’ to augment the efficacy of immunotherapeutics (147, 148). This timing corresponds to a time window of psychological modulation that coincides with the optimal immune response, in order to achieve the best effects on treatment outcomes. This kind of timing is a typical example of the convergence of the fields of psychoneuroimmunology and oncology, which for example enables a paradigm shift to integrative and temporally coordinated cancer treatment (147). Overall, this stratified framework should be positioned as a conceptual hypothesis to guide future research rather than as an established protocol for clinical decision-making. Each of its matching recommendations requires independent prospective validation, particularly to establish whether genotype-stratified psychological interventions genuinely produce between-group differences in immunological and, ultimately, oncological endpoints (**Table 3**). Until such evidence is available, the framework should not be used to guide prescribing decisions.

**Table 3:** Stratified framework of precision psychological prescriptions matched by tumor genotypes and psychological phenotypes.

Tumor molecular/clinical subtype	Corresponding psychological phenotype	Core immune microenvironment feature	First-line psychological intervention	Synergistic augmentation strategy	Reference
TP53 loss-of-function mutation	High-inflammatory anxiety phenotype, emotional irritability, exaggerated stress response	Sustained IL-6 elevation, hyperactivated NF-κB pathway, increased Treg infiltration	Mindfulness-based stress reduction (MBSR), 5 standardized sessions per week	Combined β-adrenergic blockade (propranolol) to suppress adrenergic signaling	(6, 44, 131)
KRAS activating mutation	Pathological worry, cognitive inflexibility, persistently elevated cortisol	Increased oxidative stress, accumulated DNA damage, decreased NK cell activity	Cognitive behavioral therapy (CBT) focusing on cognitive restructuring and flexibility training	Combined antioxidant nutritional support to attenuate oxidative damage	(7, 10)
High-inflammatory & high-anxiety composite phenotype	Generalized anxiety with somatic symptoms, low stress threshold	Elevated systemic CRP, imbalanced Treg/Th17 ratio, prominent immunosuppression	Integrated MBSR + CBT intervention with daily short-term mindfulness practice	Combined non-steroidal anti-inflammatory drugs for synergistic inflammation control	(80)
Low-inflammatory & insufficient social support phenotype	Strong loneliness, sparse social network, inhibited emotional expression	Insufficient CD8^+^ T cell tumor infiltration, downregulated ICAM-1 expression	Peer support groups + social skills training to strengthen social connectivity	Combined oxytocin-associated social intervention to enhance immune infiltration	(102, 149, 150)
High post-traumatic growth (PTG) propensity phenotype	Strong cognitive restructuring ability, dominant positive affect, adaptive coping	Higher baseline NK cell activity, stronger immune homeostasis maintenance	Expressive writing intervention to guide meaning-making of cancer-related trauma	Regular NK cell activity monitoring as a biofeedback indicator	(137, 138, 151)
Immune checkpoint inhibitor (ICI) treatment phase	Treatment-related anxiety, anticipatory distress, fluctuating immune cycle	High T cell exhaustion level, insufficient tumor-infiltrating lymphocyte density	Intensified psychological intervention from 48 h before to 72 h after ICI administration	Time-aligned with immunotherapy cycle to amplify immune cycle effect	(147)

This table proposes a stratified construct for personalized psycho-oncological care that is intended to inform the design of prospective trials, integrating tumor genomic profiles, psychological phenotyping and immune status. The matching relationships shown here currently constitute hypotheses awaiting validation, are not clinically actionable, and should not be used directly to guide clinical prescribing.

### 5.3 Supporting evidence, preliminary signals and evidence gaps

Emerging empirical evidence points to the potential of precision psychological interventions in oncology and provides a rationale for exploring their integration into personalized cancer care, while falling short of supporting their immediate implementation. Emerging clinical evidence links psychological status to immunotherapy efficacy. A recent prospective study in 2023 showed that baseline emotional well-being in advanced cancer patients is significantly associated with clinical responses to immune checkpoint inhibitors. Lower psychological distress correlated with favorable systemic immune profiles, such as reduced soluble CTLA-4, demonstrating the potential of psychological care to complement PD-1/PD-L1 treatments and enhance clinical outcomes (152).

Mechanistic studies have also helped to clarify the biology underpinning these effects, including the brain-bone marrow axis. Chronic stress triggers epigenetic rewiring of hematopoietic stem cells (HSCs) with increased trimethylation of H3K4me3 on the promoters of myeloid differentiation genes, driving a balance of hematopoiesis toward pro-tumorigenic myeloid pathways. Strikingly, the epigenetic marks were reversed after 4 weeks of PEEW(Prolonged Emotional Expressive Writing), increasing the number of anti-tumor subsets of neutrophils, including Ly6G+ (42). This epigenetic plasticity highlights the strong effect of psychological states on the hematological and immune systems and provides a mechanistic basis for the incorporation of behavioral interventions in cancer treatment (153).

The development of digital phenotyping has led to the development of new biomarkers for the early detection of tumor recurrence and psychological deterioration. Voice tremor, the number of contacts per day on social networks, etc., are examples of parameters that are sensitive indicators of the patient’s state. Within 1 month, the sensitivity and specificity of abnormal tumor recurrence signals (such as elevation of so-called circulating tumor DNA, ctDNA) are 78% and 92% respectively when there is a concurrent decrease of >20% in both metrics. The high predictive power allows timely clinical interventions, and it is a good example of the convergence of digital health technologies and psycho-oncology for personalized monitoring (154).

Taken together, these advances provide an emerging—but still preliminary—evidence base for precision psychological interventions, encompassing immunological modulation, epigenetic reprogramming and digital biomarker surveillance. The constituent findings differ substantially in study design, sample size and endpoint type, and most remain to be replicated in independent, adequately powered cohorts. They represent a paradigm shift from symptom management to biobehavioral remodeling, and clearly emphasize the need for multidisciplinary approaches involving molecular oncology, psychoneuroimmunology, and digital health to enable optimal cancer rehabilitation and therapeutic responsiveness.

**Figure 3** shows stratified precision psychological interventions synergizing with immune checkpoint inhibitors under different tumor genotypes. (A) In TP53-mutant tumors, NF-κB hyperactivation and elevated IL-6 levels sustain a pro-tumorigenic state marked by anxiety-related inflammation and excessive Treg infiltration. When MBSR and β-blockers are used in combination, they serve to restrain NF-κB signaling, normalize the Treg/Th17 ratio, and boost the efficacy of PD-1 therapy. (B) KRAS-mutant tumors are characterized by cortisol overproduction and ROS accumulation, which in turn drive cognitive anxiety and suppress NK cell activity. Through the application of CBT, oxidative stress can be alleviated and NK cell cytotoxicity recovered. (C) When intensive psychological intervention is delivered within the 48-72-hour window following ICI administration, it acts to increase intratumoral TIL infiltration and potentiate the full antitumor immune cycle. Abbreviations: TP53, tumor protein p53; KRAS, Kirsten rat sarcoma viral oncogene homolog; NF-κB, nuclear factor κB; IL-6, interleukin-6; TME, tumor microenvironment; Tregs, regulatory T cells; MBSR, mindfulness-based stress reduction; CBT, cognitive behavioral therapy; ROS, reactive oxygen species; NK, natural killer; ICI, immune checkpoint inhibitor; TILs, tumor-infiltrating lymphocytes.

**Figure 3:**
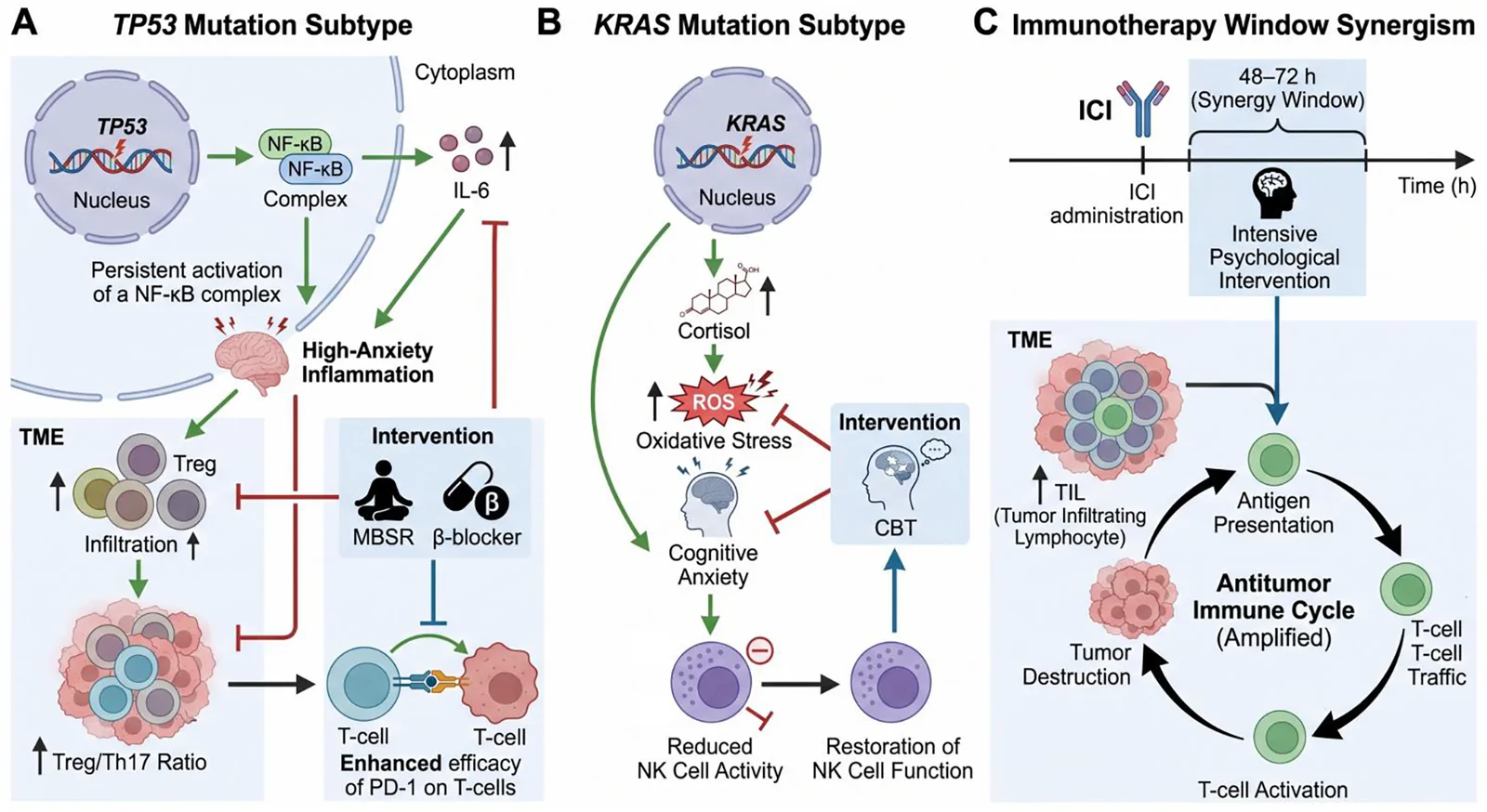
Genotype-stratified precision psychological interventions synergize with immune checkpoint inhibitors via distinct molecular pathways. **(A)** In TP53-mutant tumors, MBSR combined with β-blockers inhibits NF-κB signaling, normalizes the Treg/Th17 ratio, and enhances PD-1 therapy efficacy. **(B)** In KRAS-mutant tumors, CBT reduces cortisol levels and oxidative stress, restoring NK cell activity. **(C)** Intensive psychological intervention delivered within 48-72 hours after ICI administration increases intratumoral TIL infiltration and potentiates the full antitumor immune cycle.

## 6 Conclusions and perspectives

From an expert’s point of view, the paradigm of cancer psychological rehabilitation goes beyond symptom management and suggests a new definition of therapeutic endpoint – the measurable biological indicators, such as the “immune reconstruction index” and “tumor dormancy maintenance capacity. “ The concept departs from the traditional view of oncology and psychosocial care as a collection of separate elements, moving instead toward an integrative account in which psychological health is plausibly linked to immune competence and tumor control. We emphasize the word plausibly: the available human data are largely observational and rely on surrogate endpoints, and both the direction and the magnitude of these links remain to be established. This integrative framework must, however, accommodate the growing recognition that tumors arising from the same organ can behave as biologically distinct diseases at the molecular level, with divergent toxicity profiles, symptom trajectories, and supportive care needs.

The dynamic biobehavioral model is an important scaffolding model, as it highlights psychological carcinogenic factors that can be modified through interventions: chronic stress, disruption of circadian rhythm, and social isolation. These factors can create a vicious “stress-inflammation-immune evasion” cycle that may be interrupted by real-time biofeedback systems. It should be stressed that this cycle is not a unidirectional pathway: tumor burden and treatment-related inflammation can also aggravate psychological distress in the reverse direction, so that any evaluation of interventions targeting this cycle must measure effects in both directions. The mechanistic insight is relevant to both psychoneuroimmunology and clinical oncology and highlights the need to address psychosocial determinants to control systemic inflammation and reestablish immune surveillance. Such dynamic feedback loops hold great promise for clinical use, but will need careful validation in order to reconcile molecular, behavioral science, and clinical outcome findings. Importantly, the magnitude and trajectory of these stress-induced immune alterations are likely modulated by underlying tumor molecular heterogeneity, reinforcing the need for subtype-stratified approaches in both research and practice.

Furthermore, personalizing psychological prescriptions depending on the genetic characteristics of the tumor, such as TP53 and KRAS mutations, and psychological traits, such as a tendency toward post-traumatic growth, represents an extension of precision medicine to psycho-oncology. This personalized approach can serve as a means to enhance the immune response against tumor cells, especially when combined with other immune therapies such as checkpoint inhibitors. This integration of genomic and psychological information can lead to a more comprehensive understanding of patient heterogeneity, which can guide personalized interventions that maximize therapeutic benefit and reduce adverse effects. This requires, however, a consideration of the intricate relationships among genetic mutations, epigenetic regulation, and psychosocial factors and requires multi-disciplinary approaches and sophisticated analytical methods. It must be emphasized that this genotype- and phenotype-informed prescribing framework remains at the hypothesis stage. Until prospective randomized controlled evidence is available, its value should be confined to that of a research hypothesis rather than a practice recommendation.

This molecularly informed perspective also has profound implications for the future architecture of oncological rehabilitation research. Historically, rehabilitation protocols and PROMs instruments have been organized primarily by tumor site or treatment modality. Yet, as the foregoing subtype-specific examples illustrate, this anatomy-centric taxonomy no longer suffices. We therefore advocate that future PROMs development should incorporate mutation- or subtype-specific symptom modules, that biomarker interpretation be calibrated to molecular context, and that the interplay between psychosocial stress and tumor biology be examined through a genotypic lens. To translate these concepts into practice, future research should systematically incorporate molecular subtype stratification as a core design variable in rehabilitation trials and PROMs validation studies. While this vision awaits empirical validation, the evidence reviewed here provides a mechanistic rationale and an initial roadmap toward a personalized, biologically informed approach to cancer rehabilitation.

In the future, the development of a multimodal and dynamic “psychological-immune-tumor” atlas is a visionary action that can map the temporal and spatial interactions in this triad. The complete framework might make it easier to identify new “psych-oncological” biomarkers and therapeutic targets, thus speeding up the development of “psych-oncological” pharmacotherapies which act via epigenetic mechanisms. At the same time, the recent emergence of the metaverse and immersive biofeedback systems provides a novel method for delivering personalized, engaging and scalable interventions, overcoming geographical and socioeconomic challenges. The development of these technologies has the potential to make brain-body integrative therapies accessible and available to anyone, which is a broad goal of low-cost, high-benefit rehabilitation for cancer survivors. Crucially, such platforms could be designed to incorporate subtype-specific PROMs modules and adaptive intervention algorithms, further enabling the molecularly-stratified supportive care envisioned above.

By weaving together these diverse perspectives, this review puts forward a testable hypothesis framework that could reshape current clinical guidelines—including those issued by the NCCN—by embedding precision psychological immuno-oncology within standard cancer rehabilitation protocols. The brain-body integrative model proposed here would not only expand the range of therapeutic options available against cancer, but also equip survivors with sustainable approaches to improving both quality of life and long-term prognosis. However, achieving such a vision requires a coordinated approach with regard to translational research, strong clinical trials, and interdisciplinary education to facilitate the translation of new knowledge. In conclusion, this biologically-oriented, multi-faceted approach to cancer psychological rehabilitation represents a new paradigm in oncology that recognizes and utilizes the intricate relationship among mind, immune system and the biology of the tumor.
